# Expert perspectives for the pharmacist on facilitating and improving the use of albumin in cirrhosis

**DOI:** 10.1093/ajhp/zxad070

**Published:** 2023-04-04

**Authors:** Marcelo Kugelmas, Michelle Loftus, Emily J Owen, Hani Wadei, Sammy Saab

**Affiliations:** South Denver Gastroenterology, Englewood, CO, USA; Division of Hospital Medicine, North Shore University Hospital, Hempstead, NY, and Donald and Barbara Zucker School of Medicine at Hofstra/Northwell, Hempstead, NY, USA; Critical Care, Surgical Burn Trauma Intensive Care Unit, Department of Pharmacy, Barnes-Jewish Hospital, St. Louis, MO, USA; Department of Transplantation, Mayo Clinic, Jacksonville, FL, USA; Department of Internal Medicine and Surgery, David Geffen School of Medicine, UCLA, Los Angeles, CA, USA

**Keywords:** albumin, ascites, cirrhosis, hepatorenal syndrome, spontaneous bacterial peritonitis, terlipressin

## Abstract

**Purpose:**

Albumin, the most abundant and arguably most important protein in the human body, plays a unique role in decompensated cirrhosis because its structure and function are quantitatively and qualitatively affected. A literature review was performed to provide insights into albumin use. The manuscript was developed using a multidisciplinary approach; 2 hepatologists, a nephrologist, a hospitalist, and a pharmacist, who are all members of or work closely with the Chronic Liver Disease Foundation, collaborated to write this expert perspective review.

**Summary:**

Cirrhosis represents the potential end in the spectrum of all chronic liver diseases. Decompensated cirrhosis, defined by the overt manifestation of liver failure (eg, ascites, hepatic encephalopathy, variceal bleeding), is the inflection point associated with increased mortality. Human serum albumin (HSA) infusion serves an important role in the treatment of advanced liver disease. The benefits of HSA administration in patients with cirrhosis are widely accepted, and its use has been advocated by several professional societies. However, inappropriate HSA use can lead to significant adverse patient events. This paper discusses the rationale for the administration of HSA in the treatment of complications of cirrhosis, analyzes the data on the use of HSA in cirrhosis, and streamlines practical recommendations set forth in published guidance.

**Conclusion:**

Use of HSA in clinical practice needs to be improved. The objective of this paper is to empower pharmacists to facilitate and improve the use of HSA in patients with cirrhosis at their practice sites.

**Key Points**

- The American Association for the Study of Liver Diseases provides guidance on human serum albumin (HSA) dosing and administration for ascites, hepatorenal syndrome–acute kidney injury, and spontaneous bacterial peritonitis, but, ultimately, treatment requires adjustment based on individual patient status and HSA administration cannot be indefinite.
- Signs of cardiopulmonary dysfunction and fluid status (eg, blood pressure, heart rate, oxygenation, escalating oxygen requirements, respiratory rate, development of peripheral edema, and renal function) should be assessed after each dose of HSA, and the dose and administration should be adjusted accordingly.
- Correcting HSA based on fluid status is more important than achieving goal albumin levels.

Cirrhosis is defined as the histological development of regenerative nodules surrounded by fibrous bands in response to chronic liver injury that leads to portal hypertension and end-stage liver disease. ^1^

Resulting from numerous etiologies (eg, chronic viral infection, alcohol- and non–alcohol-related liver disease, hereditary diseases), cirrhosis represents the end in the spectrum of chronic liver diseases (CLDs)^2^ and afflicts 10% to 20% of patients with CLD within 10 to 20 years of diagnosis.^3^ In 2018, the Centers for Disease Control and Prevention estimated that 4.5 million adults in the US had been diagnosed with CLD.^4^ Liver disease accounts for approximately 2 million deaths each year worldwide, half of which are attributed to cirrhosis. The natural history of cirrhosis is characterized by compensated cirrhosis, a period of disease associated with normal or nearly normal liver function, followed by decompensated cirrhosis, when clinical signs and symptoms of hepatic dysfunction such as jaundice, ascites, hepatic encephalopathy (HE), hepatorenal syndrome (HRS), and variceal hemorrhage are observed.

Albumin is the most abundant and arguably most important plasma protein in the human body.^5^ As liver disease progresses to decompensated cirrhosis, quantitative, qualitative, and functional changes to albumin occur, and supplementation with human serum albumin (HSA), a colloid preparation, is beneficial.^6,7^ Throughout the present paper, serum albumin levels will be referred to as albumin and human serum albumin as a drug product will be referred to as HSA. HSA supplementation dates back to the 1940s.^8^ The benefits of HSA use in CLD have been demonstrated in numerous studies,^9-16^ and guidance from the American Association for the Study of Liver Diseases (AASLD) provides direction on HSA administration in cirrhosis.^9^ The most current and well-established indications for the use of HSA in cirrhosis pertain to conditions characterized by an acute worsening of effective hypovolemia,^17^ such as ascites, postparacentesis circulatory dysfunction (PPCD), spontaneous bacterial peritonitis (SBP), and HRS–acute kidney injury (HRS-AKI).^9^

HSA is often misused and, as such, has been described in the literature as “a much abused and expensive drug,”^18^ “erroneously prescribed,”^19^ and a drug for which the “use in clinical settings continues to generate controversy,”^20^ while “periodic shortages and the high cost of HSA have compelled many hospitals to develop guidance regarding HSA administration.”^20^ A review of the literature indicated that HSA misuse has been studied in Italy, and some of these data are summarized in Table 1. The following clinical practice gaps are associated with HSA use and may contribute to its misuse: (1) delayed or prolonged administration in the clinical course; (2) unclear serum albumin goal levels and speed of correction; (3) lack of recognition of the benefits of HSA; (4) uncertainty regarding the specialist responsible for prescribing HSA (eg, hepatologist, nephrologist, hospitalist); and (5) use beyond approved indications.

**Table 1.** Identified Trends in Human Serum Albumin Misuse in Italy

Study	Aim	Findings
Finelli et al^19^ (2001)	This retrospective study reviewed HSA prescriptions and indications in 4,122 patients hospitalized in internal medicine wards in the years 1996, 1998, and 1999, before and after the ad hoc regulations issued in 1997 by the Italian Ministry of Health.^a^	• In the study, 161 patients (91%) administered HSA had serum albumin values of <3.5 g/dL, while in only 36% of patients were these values <2.5 g/dL. • The most common daily dosage was 10 g of HSA, corresponding to 50 mL of 20% HSA. • In the majority of cases, HSA was erroneously prescribed, often to enhance diuretic efficacy or in protein energy malnutrition. • The new Italian Ministry of Health recommendations did not influence how albumin was prescribed.
Casuccio et al^21^ (2015)	Investigators assessed the appropriateness of requests for HSA using current guidelines and evaluated physicians’ compliance with this protocol. An experimental, pharmacist-driven process for daily orders of HSA was activated over a period of 1 month.	• A total of 126 forms were collected. • The mean serum albumin and protein levels of patients in different wards were close to normal (2.5 and 5 g/dL, respectively). • The HSA doses requested by the various wards were 2 to 7 times higher than expected. • According to current guidelines, 83.3% of requests across different wards were appropriate; 45% of orders from the ICU were for inappropriate indications.

Abbreviations: HSA, human serum albumin; ICU, intensive care unit.

^a^In 1997, the Italian Ministry of Health released an “ad hoc” protocol limiting the use of human serum albumin to only patients with a serum albumin concentration of <2.5 g/dL, anasarcatic conditions (a generalized accumulation of fluid in the interstitial space), or when other colloids are contraindicated.

Knowledge of HSA indications and supporting evidence is paramount in allowing prescribers to make safe and effective clinical and economic decisions.^22^ As medication therapy experts in the healthcare system, pharmacists are essential members of the multidisciplinary team for medication decision-making.^23^ Data on pharmacist-led interventions regarding HSA prescribing have demonstrated beneficial effects, including reductions in inappropriate use, lower costs, and more efficient integration of multidisciplinary evidence-based guidance into practice.^24-26^ As such, pharmacists can play a key role in overcoming the above practice gaps. This paper will discuss the practice gaps and rationale for using HSA in the treatment of patients with cirrhosis, analyze data on the current use of HSA in cirrhosis, and streamline the practical recommendations set forth in published guidance. The objective is to empower pharmacists to facilitate and improve the use of HSA for cirrhosis across their multidisciplinary teams.

## Albumin physiology

Albumin is the most abundant protein in the blood (normal value of 3.5-5.0 g/dL; accounting for about 50% of all plasma protein) and extracellular fluids and is the main modulator of fluid distribution between body compartments.^6,17,27-29^ The 2 essential physiological functions of albumin are to contribute to colloid osmotic pressure and to aid in the transportation, distribution, and metabolism of endogenous and exogenous molecules.^27^ Albumin is considered a multifunctional protein because it exhibits “extraordinary” ligand-binding capacity, serves as a valuable biomarker for an array of diseases, and is used as a “tool” to expand total plasma volume when treating many diseases, including CLD.^27-29^

## Cirrhosis and albumin pathophysiology

By understanding how albumin malfunctions in cirrhosis, pharmacists can garner a better understanding of, and subsequently educate the multidisciplinary team on, why HSA is indicated in cirrhosis. In cirrhosis, albumin undergoes quantitative and, to a greater extent, qualitative changes.^17^ Under normal conditions, 10 to 15 g of albumin is synthesized daily within hepatocytes; in advanced liver disease, hepatocyte loss and ongoing inflammation result in decreased albumin synthesis.^30^ Therefore, quantitatively, serum albumin reductions are common in patients with cirrhosis.^17,31^ Qualitatively, molecular abnormalities of albumin also occur in patients with cirrhosis (eg, oxidation, reduced binding capacity, and development of homodimers), which affect the functional properties of the protein.^17^ As a result, the concentration of functionally intact albumin is reduced to an even greater extent.^17^ One example of the consequences of reduced albumin concentrations can be observed with bile acids. Albumin exclusively binds bile acids for delivery to hepatocytes for processing and elimination; in cirrhosis, albumin loss contributes to increased serum bile acid concentrations.^6^

Clinical outcomes in cirrhosis have been shown to correlate with reduced albumin concentrations. The Child-Pugh scoring system (also known as the Child-Turcotte-Pugh score) is used as a guide to predict mortality in patients with cirrhosis. The scoring system divides patients into 3 categories: (A) patients with good hepatic function (5 to 6 points); (B) those with moderately impaired hepatic function (7 to 9 points); and (C) those with advanced hepatic dysfunction (10 to 15 points). Albumin levels are considered to be one of the essential clinical measures of the synthetic function of the liver and, as such, are part of this scoring system; albumin levels above 3.5 g/dL are equal to 1 point, 2.8 to 3.5 g/dL are equal to 2 points, and less than 2.8 g/dL are equal to 3 points.^32^

In healthy individuals, a small proportion of albumin exists as mixed disulfide compounds known as human nonmercaptalbumin 1 (HNA1), and an even smaller fraction is found in a highly oxidized form known as human nonmercaptalbumin 2 (HNA2).^33^ In advanced liver disease, increases are observed in the abundance of the irreversibly oxidized form of albumin known as human nonmercaptalbumin (HNA).^34^ One study found that the plasma levels of HNA2 are closely related to survival in decompensated cirrhosis and acute-on-chronic liver failure (ACLF), with a strong correlation of HNA2 with 30- and 90-day mortality. Increased concentrations of HNA1 and HNA2 correlate closely with Model for End-Stage Liver Disease score, bilirubin concentration, international normalized ratio, and C-reactive protein concentration.^33^ In a larger study (N = 2,376 patients hospitalized for cirrhosis but without HE), decreased serum albumin levels were potentially associated with higher risk of overt HE (odds ratio [OR] = 0.878, 95% confidence interval [CI] = 0.834-0.924) and death from overt HE (OR = 0.864, 95% CI = 0.771-0.967).^35^

## HSA as a drug

Medicinal HSA is a parental colloid derived from human plasma. Two formulations are available that differ in albumin concentration: 5% and 25% HSA. In general terms, 25% HSA is the therapeutic choice when fluid is restricted or in cases of oncotic deficiency,^27^ which is common in patients with cirrhosis who are prone to developing edema from infusions with a large intravenous volume, thereby making it the HSA formulation that should be used in most circumstances for patients with cirrhosis. Use of 5% HSA is more common in rapid response situations that necessitate large volume replacement to restore hemodynamics and counteract volume loss. For cirrhotic patients with hypovolemia, 5% HSA may be an alternative option as a volume expander, usually used as a replacement for or alternating with crystalloids. However, minimal evidence exists to support this practice outside of a small pilot study of hypoalbuminic patients requiring volume resuscitation.^36^ Because 25% HSA is the most commonly utilized formulation in cirrhotic patients, 5% HSA will not be discussed further, with the exception of the information that follows, which is of utmost importance to pharmacists. There has been a shortage of 5% HSA in the US since the early 1990s. Because of this shortage, hospital pharmacists have resorted to preparing 5% HSA by diluting 25% HSA with 0.9% sodium chloride or, when sodium load is a concern, 5% dextrose. Sterile water should never be used as the diluent, as it reduces the osmolarity (tonicity) of the HSA solution, which may cause hemolysis in recipients (as indicated by 10 case reports in the literature).^37,38^

To understand the benefits of HSA as a treatment in cirrhosis, it is important for pharmacists to understand the circulatory abnormalities that occur in cirrhosis leading to a hyperdynamic circulatory state. In advanced cirrhosis, disruption of the liver architecture leads to portal hypertension, which reduces portal blood flow. To compensate, concentrations of vasodilating factors (eg, carbon monoxide, nitric oxide) increase and reactivity to vasoconstrictors decreases, causing primary vasodilation of the splanchnic arterial circulation. Blood pools in the splanchnic circulation, causing ascites and hypovolemia. In response, the activity of vasoconstrictor factors (namely the renin-angiotensin-aldosterone system, sympathetic nervous system, and nonosmotic arginine-vasopressin [antidiuretic hormone] secretion) increases in an effort to increase systemic vascular resistance. Decrease in renal blood flow leads to renal sodium and water retention with subsequent plasma volume expansion, worsening edema and ascites. Nonosmotic release of vasopressin also magnifies water retention from the distal nephron, leading to hyponatremia. Despite the decrease in renal blood flow, the glomerular filtration rate (GFR) remains stable due to the ability of the kidney to maintain a steady GFR across a wide range of renal blood flow rates, which is known as renal autoregulation. Progression of liver disease, cirrhotic cardiomyopathy, and systemic inflammation (eg, sepsis) are among the factors that can overwhelm renal autoregulation, leading to worsening GFR and a rise in serum creatinine levels (otherwise known as HRS).^6,17,39^ Exogenously administered 25% HSA increases the oncotic pressure of the intravascular system, increases fluid mobilization from the interstitial space, helps restore systemic circulation, increases renal blood flow, and restores GFR.^40,41^

## Liver-related indications for HSA administration

Although overarching clinical practice guidance and guidelines on HSA use are lacking, specialty practice guidelines are available.^42^ While many uses of HSA are considered “inappropriate” and may need to be curbed, the most current and well-established indications for the use of HSA in cirrhosis pertain to conditions characterized by an acute worsening of effective hypovolemia,^17^ such as ascites, PPCD, SBP, and HRS-AKI.^9^ As such, pharmacists can ensure that these indications are added as “appropriate” on any institutional guidelines that are developed and become familiar with the situations listed in Table 2 where HSA administration may provide benefit. In the US, the principal guidance on liver-related indications for HSA use is available from the AASLD.^9^ Data from a systematic review have demonstrated that, in hospitals, education-based interventions, led by pharmacists, are effective in increasing guideline compliance and encouraging appropriate use of medications.^44^ Cirrhosis-related conditions and the rationale for HSA use are summarized in the text below, and HSA indications as well as dosing and administration recommendations are summarized in Table 2. This information is intended for pharmacists to use as a resource when educating the multidisciplinary team on HSA use and developing institution-specific guideline recommendations.

**Table 2.** AASLD Guidance Recommendations on the Use of Human Serum Albumin in Cirrhosis

Condition	Indication	Treatment dosage(s) and administration
Grade 3 ascites^9^	Administer HSA if LVP is >5 L to mitigate the risk of PPCD; the risk of PPCD may increase with >8 L of fluid evacuated in a single session	• 25% HSA: 5-10 g (8 g is usually quoted; maximum dose, 50 g) for every liter of ascites removed during and after paracentesis, which is a one-time intervention; discontinue administration if an infusion-related AE occurs • HSA is usually rounded to the nearest vial size (50 mL [12.5 g] and 100 mL [25 g])
HRS-AKI diagnosis^9^	HSA challenge is recommended in patients with stage 2 or 3 AKI and elevated SCr that becomes further elevated despite risk factor management^a^	Administer 25% HSA 1 g/kg IV daily (maximum dose, 100 g/day; maximum rate, 1-2 mL/min) until adequate volume is achieved (as indicated by improvement in hemodynamic parameters and renal function) or a maximum of 2 days
HRS-AKI treatment	HSA in combination with a vasoconstrictor is indicated in patients who meet the criteria for HRS-AKI^b^	First choice: terlipressin + 25% HSA^9^ • Days 1 to 3: administer terlipressin 0.85 mg (1 vial) IV every 6 hours^43,^^c^ • Day 4: assess SCr vs baseline^43,^^c^: • If SCr has decreased by at least 30% from baseline, continue terlipressin 0.85 mg (1 vial) IV every 6 hours • If SCr has decreased by less than 30% from baseline, the dose may be increased to terlipressin 1.7 mg (2 vials) IV every 6 hours • Coadminister HSA 1 g/kg IV on day 1 of therapy followed by 40-50 g/day, for the duration of therapy^9^
		Second choice: norepinephrine + 25% HSA • Start norepinephrine, via continuous IV infusion, at 0.5 mg/h to achieve an increase in mean arterial pressure of at least 10 mm Hg or an increase in urine output of >200 mL/4 h; if at least one of these goals is not achieved, increase the dose every 4 hours in increments of 0.5 mg/h, up to a maximum of 3 mg/h • Response to norepinephrine is defined by a decrease in SCr to <1.5 mg/dL or a return to within 0.3 mg/dL of baseline over a maximum of 14 days; in patients whose SCr remains at or above the pretreatment level over 4 days with the maximum tolerated dose of the vasoconstrictor, therapy may be discontinued • Coadminister 25% HSA to maintain a central venous pressure between 4 and 10 mm Hg
		Third choice: midodrine/octreotide + 25% HSA^d^ • Administer oral midodrine 5-15 mg by mouth every 8 hours in combination with octreotide 100-200 μg subcutaneously every 8 hours or 50 μg/h IV • Maintain midodrine/octreotide until SCr returns to baseline (up to 14 days), which may be extended in certain cases; in patients whose SCr remains at or above the pretreatment level over 4 days with the maximum tolerated doses of midodrine/octreotide, therapy may be discontinued • Coadminister 25% HSA 25 g IV twice daily for 6 doses, with daily re-evaluation and decision-making according to patient status
SBP	Initiate treatment once the diagnosis of SBP is established (ascitic fluid has a PMN leukocyte count of >250/μL)	• Start IV antibiotics empirically in all patients with an ascites/pleural fluid PMN count of >250/μL; first-line empirical antibiotic therapy for community-acquired SBP/SBE is IV third-generation cephalosporin (eg, cefotaxime 2 g IV every 12 hours) • Response to empirical antibiotic therapy may be assessed by repeating diagnostic paracentesis/thoracentesis 2 days after initiation; a decrease in fluid PMN of <25% from baseline indicates lack of response and should lead to broadening of antibiotic coverage and further evaluation to rule out secondary bacterial peritonitis • Coadminister 25% HSA, 1.5 g/kg at day 1 and 1 g/kg at day 3

Abbreviations: AASLD, American Association for the Study of Liver Diseases; AE, adverse event; AKI, acute kidney injury; HRS-AKI, hepatorenal syndrome–acute kidney injury; HSA, human serum albumin; IV, intravenous; LVP, large volume paracentesis; PMN, polymorphonuclear; PPCD, postparacentesis circulatory dysfunction; SBE, spontaneous bacterial empyema; SBP, spontaneous bacteria peritonitis; SCr, serum creatinine.

^a^Risk factor management includes withdrawal of nephrotoxic drugs, reduction or withdrawal of diuretics, detection and treatment of infections if present, and volume replacement (in the case of severe volume depletion) using 5% human serum albumin or crystalloids, preferentially balanced, initially.

^b^Cirrhosis with ascites; diagnosis of acute kidney injury according to International Club of Ascites–Acute

Kidney Injury criteria (increase in serum creatinine of ≥0.3 mg/dL from baseline within 48 hours or a percent increase in serum creatinine of ≥50%, which is known or presumed to have occurred within the preceding 7 days); no response after 2 consecutive days of diuretic withdrawal and plasma volume expansion with human serum albumin infusion (1 g/kg body weight per day); absence of shock; no current or recent use of nephrotoxic drugs (nonsteroidal anti-inflammatory drugs, aminoglycosides, or iodinated contrast media); and no signs of structural kidney injury, as indicated by proteinuria (>500 mg/day), microhematuria (>50 red blood cells per high-power field), and/or abnormal renal ultrasonography.

^c^The AASLD guidance was published before approval by the Food and Drug Administration of terlipressin. For the purposes of this manuscript, the recommended dose and administration for terlipressin are based on the Food and Drug Administration–approved prescribing information.

^d^AASLD warns that the efficacy of this treatment regimen is low.

### Ascites and large volume paracentesis (LVP).

Ascites is fluid accumulation in the abdominal cavity, which is typically the first event associated with decompensated cirrhosis.^9^ Ascites carries a 1-year mortality rate of 15% and a 5-year mortality rate of 44%; mortality rates increase as complications of decompensated cirrhosis increase. A serum albumin ascites protein gradient greater than 1.1 is used to establish portal hypertension as the cause of ascites with cirrhosis, which is the most common cause of portal hypertension. Ascites is graded according to the amount of fluid accumulated in the abdominal cavity, with grade 1 ascites detectable by ultrasound, which is considered mild; grade 2 ascites causing moderate symmetrical distension of the abdomen, which is considered moderate; and grade 3 ascites causing marked and painful abdominal distension, which is considered large, massive, or tense.^43^

Grade 3 ascites (also known as diuretic-resistant ascites) is often refractory to treatment, meaning that the abdominal fluid cannot be mobilized with dietary sodium restriction and diuretic therapy. LVP, a procedure to drain ascitic fluid, is indicated, but excessive removal (>5 L)^13^ can cause a rapid and significant decrease in intra-abdominal pressure, thereby increasing cardiac return. In response, cardiac output increases and peripheral resistance decreases, ultimately leading to hypovolemia and increased plasma renin activity, which is already an issue in decompensated patients.^29^ This event, termed PPCD, leads to the faster re-accumulation of ascites, hyponatremia, HE, renal impairment, HRS, and, in some cases, death.^13,17^ HSA infusions reduce the incidence of PPCD in patients with grade 3 ascites,^29,30^ and, according to a meta-analysis, also result in greater reductions in morbidity and mortality compared to alternative plasma expanders (eg, artificial colloids and vasoconstrictors).^29^ The AASLD recommends HSA as the treatment of choice in grade 3 ascites to reduce the risk of PPCD when LVP fluid removal exceeds 5 L (Table 2).^13^

### HRS-AKI.

HRS-AKI is functional, progressive kidney failure that occurs via the mechanisms described in the above section “HSA treatment” and occurs in patients with cirrhosis.^10,15,45^

AKI is diagnosed by an increase in serum creatinine concentration of ≥0.3 mg/dL within 48 hours or a 50% or greater increase in serum creatinine levels that is known or presumed to have occurred within the preceding 7 days, but HRS cannot be confirmed until other causes of AKI have been excluded and the patient meets HRS criteria (Table 3).^9^ HRS-AKI is potentially reversible with treatment; without treatment, the consequences of HRS-AKI include irreversible renal failure followed by appreciably increased risk of death, with mortality rates approaching 100% 3 months after diagnosis.^10,48^ As such, once HRS-AKI is diagnosed, correction of underlying conditions and rapid treatment initiation are essential.^9^ Liver transplantation is the optimal treatment for patients with HRS-AKI because it corrects the underlying liver failure, but this is rarely realized because of many factors (eg, organ availability, patient contraindications to transplantation).^10^ Renal replacement therapy (ie, dialysis) may bridge patients to liver transplants, but the outcomes are poor.^10^ Therefore, clinicians rely on pharmacological therapy to improve outcomes in these patients, with such therapy involving 2 components: volume expansion and vasoconstriction.

**Table 3.** Terlipressin US Package Insert Recommendations^46^

Prescribing parameter	Description
Boxed warning	**Warning: serious or fatal respiratory failure.** Terlipressin may cause serious or fatal respiratory failure. Patients with volume overload or with ACLF grade 3^a^ are at increased risk. Assess oxygen saturation (eg, Spo_2_) before initiating terlipressin. Do not initiate terlipressin in patients experiencing hypoxia (eg, Spo_2_ of <90%) until oxygenation improve. Monitor patients for hypoxia using continuous pulse oximetry during treatment and discontinue terlipressin if Spo_2_ decreases below 90%.
Indications	To improve kidney function in adults with hepatorenal syndrome with rapid reduction in kidney function
Contraindications	In patients experiencing hypoxia or worsening respiratory symptoms and in patients with ongoing coronary, peripheral, or mesenteric ischemia
Warnings and precautions	**Serious or fatal respiratory failure.** Monitor patients for changes in respiratory status using pulse oximetry and regular clinical assessments. Actively manage intravascular volume overload and adjust terlipressin therapy as appropriate.
	**Ineligibility for liver transplantation.** Terlipressin-related adverse reactions may make a patient ineligible for liver transplantation, if listed.
	**Ischemic events.** Terlipressin is a vasoconstrictor and can cause ischemic events (cardiac, peripheral, or mesenteric) that may require dose interruption or discontinuation.
	**Embryo-fetal toxicity.** Terlipressin may cause fetal harm when used during pregnancy. Advise females of reproductive potential of the potential hazard to the fetus.
Adverse reactions	The most common adverse reactions (≥10%) include abdominal pain, nausea, respiratory failure, diarrhea, and dyspnea.

Abbreviation: ACLF, acute-on-chronic liver failure.

^a^For more information on acute-on-chronic liver failure and access to an online calculator, see reference ^47^.

HSA is considered a crucial volume expander for the treatment of HRS-AKI when used in combination with vasoconstrictors; the additive effects provided by vasoconstrictors and HSA infusion are thought to improve outcomes compared to either agent alone.^10,13^ In the US, until recently, the vasoconstrictive component included administration of norepinephrine (α-adrenergic agonism) or midodrine and octreotide (α-adrenergic agonism combined with inhibition of splanchnic vasodilation). Most of the data on these agents in HRS-AKI are based on small, nonrandomized studies, and these drugs are not approved by the Food and Drug Administration for HRS-AKI. In the past in the US and Canada, clinicians defaulted to the off-label use of these drugs in combination with HSA,^10^ whereas, elsewhere, terlipressin—a vasopressin analog that exerts vasoconstrictive activity via selective V1 and V2 agonism—is approved for HRS-AKI.^49^ Fortunately, in late 2022, terlipressin became the first drug to be approved in the US, in combination with hyperoncotic (25%) HSA solution, for the treatment of HRS-AKI.^46^ According to European Association for the Study of Liver Diseases^50^ and AASLD guidelines (Table 2),^9^ terlipressin, when combined with 25% HSA, is the treatment regimen of choice for HRS-AKI.

Terlipressin was demonstrated to be safe and effective for treatment of HRS-AKI in the phase 3 CONFIRM study, which analyzed 300 patients with cirrhosis and HRS-AKI (formerly termed HRS-1) and in which patients received terlipressin (n = 199) or placebo (n = 101) in a blinded manner. Patients were administered 1 mg of terlipressin or placebo intravenously for 2 minutes every 5.5 to 6.5 hours. It was strongly recommended that all patients receive HSA (1 g per kilogram of body weight to a maximum of 100 g on day 1 and 20 to 40 g per day thereafter). On day 4, patients with a serum creatinine level that had decreased by less than 30% from the baseline level after a minimum of 10 doses of terlipressin or placebo could receive 2 mg every 6 hours, except those with coronary artery disease, circulatory overload, pulmonary edema, or bronchospasm. The primary endpoint of verified HRS reversal—defined as 2 consecutive serum creatinine measurements of 1.5 mg/dL or less at least 2 hours apart up to day 14 and survival without renal replacement therapy for at least an additional 10 days of HRS—was reported in 32% of patients in the terlipressin group and 17% of patients in the placebo group (*P* = 0.006).^16^

The recommendations from the US package insert for terlipressin are detailed in Table 3.^46^ With the approval of terlipressin and the inclusion of a boxed warning for serious or fatal respiratory failure, pharmacists can play several roles in helping to ensure that dosing of 25% HSA and terlipressin is appropriate and safe for patients with HRS-AKI. First, HSA administration should be evaluated in conjunction with the provider team on a daily basis for adequate volume resuscitation and discontinued after 48 hours or adequate volume resuscitation. Patients who are volume overloaded or who have an ACLF grade of 3 or higher are at an increased risk, and pharmacists can assist the provider team with calculating the ACLF grade. Additionally, pharmacists can monitor patients’ oxygenation status on a daily basis utilizing oxygen saturation (Spo_2_) to ensure that patients’ oxygen saturation is not consistently trending down or below 90%. In the setting of decreased oxygenation (Spo_2_ of <90%), pharmacists can recommend discontinuation of terlipressin for safety.

### SBP.

In cirrhotic patients, infections account for 25% to 46% of hospitalizations because of acute decompensation events; SBP is the most common of these infections, occurring in up to 30% of hospitalized patients. SBP is defined as infection of ascitic fluid in the absence of any intra-abdominal, surgically treatable source of infection. Pathological changes in cirrhosis (eg, changes in intestinal microbiota, altered intestinal permeability, shunting from the reticulo-endothelial system in the liver) cause bacterial translocation from the intestinal lumen to the portal vein circulation, and subsequent seeding of ascites, urine, and lung alveoli occurs.^11,51^ The presence of SBP is frequently associated with significant further decline in patients with cirrhosis. An episode of SBP is typically the precursor to further decompensation events, including episodes of HE, portal hypertensive bleeding, and renal failure.^11^ Conversely, development of AKI is the main predictor of in-hospital mortality in patients with SBP.^9,14,52,53^ The rate of death in the first month following an episode of SBP is 30% and increases to 63% after the first year.^9,54^ Because SBP is frequently asymptomatic, bacterial infection should be suspected when a patient with cirrhosis deteriorates, particularly with encephalopathy, AKI, and/or jaundice. In these patients, a prompt general workup is recommended. According to AASLD guidance, diagnostic paracentesis should be performed in all patients with new-onset ascites accessible for sampling. The initial laboratory investigation of ascitic fluid should include ascitic fluid neutrophil count and ascitic fluid total protein, ascitic fluid albumin, and serum albumin concentrations.^9^

Empiric broad-spectrum antibiotic therapy should be started following diagnostic paracentesis and narrowed once the organism is isolated.^9^ Despite antibiotic therapy and resolution of infection, approximately one-third of patients with SBP develop renal failure,^52^ which is attributed to a decrease in effective arterial blood volume caused by the systemic inflammatory reaction to the infection.^52,55^ The first trial to demonstrate the benefits of plasma volume expansion with HSA in patients with cirrhosis and SBP was performed by Sort et al.^14^ Intravenous HSA (1.5 g per kilogram of body weight at diagnosis, followed by 1 g per kilogram of body weight on day 3) plus cefotaxime (n = 63), here compared with intravenous cefotaxime alone (n = 63), was demonstrated to result in significantly less kidney impairment (defined by a predetermined increase in blood urea nitrogen or serum creatinine) in hospitalized patients with cirrhosis and SBP (10% vs 33%, respectively; *P* = 0.002). In addition, 10% of patients in the cefotaxime-plus-HSA group died in the hospital as compared to 29% of patients in the cefotaxime group (*P* = 0.01). At 3 months, the mortality rate was 22% vs 41%, respectively (*P* = 0.03). Patients in the cefotaxime-plus-HSA group who demonstrated the most benefit had baseline evidence of renal dysfunction (blood urea nitrogen concentration of >30 mg/dL or creatinine concentration of >1.0 mg/dL) or severe hepatic decompensation (bilirubin concentration of >5 mg/dL).^14^ To date, HSA has been demonstrated to be the most effective plasma volume expander in these patients and is considered the standard of care.^9,11,12^ As such, the AASLD recommends use of HSA in patients with SBP (Table 3).^9^

Pharmacists can continue to play a role in antimicrobial stewardship by selecting the appropriate antibiotic based on the patient’s past microbiological history, previous antimicrobial exposure, allergies, and kidney function. They can also have an active role in ensuring that patients are given 25% HSA as a volume expander to help prevent further renal dysfunction in patients with SBP.

## Real-world expert perspectives on HSA use

From a physiological perspective, HSA is considered an ideal treatment for complications associated with CLD. However, the clinical practice gaps associated with HSA adversely affect the benefits of the colloid. The literature indicates that the high cost and periodic shortages with HSA have made its appropriate use a long-standing subject of debate.^42^ Inappropriate use for noncirrhosis indications also clouds the picture of when HSA should be used. The AASLD has acknowledged that the dosing recommendations put forth in its guidance are somewhat nonspecific because of a lack of high-quality studies in patients with conditions associated with cirrhosis.^9^ Furthermore, HSA treatment requires adjustments based on patient status, so each clinical situation is different. Orders for HSA administration cannot be indefinite; pharmacists can help ensure that durations are added to HSA orders to help promote frequent reassessment of therapy and ensure that treatment can be discontinued once a patient reaches their individual endpoints. The following sections provide real-world, multidisciplinary expert perspectives that can aid in HSA administration.

### Serum albumin levels.

It is well established that, in CLD and associated inflammation, acute phase proteins (APPs) are released. Production of certain APPs, known as positive APPs, increases. Conversely, production of other APPs, known as negative APPs, decreases. Albumin is a negative APP, and serum albumin levels decrease in cirrhosis.^56^ Although it has been well established that low serum albumin concentrations indicate poor clinical outcomes, uncertainties remain. Albumin concentrations do not correlate with albumin function, so the target for an effective albumin concentration remains undefined. There is no substantial evidence that raising serum albumin concentrations to the normal range (3.5-5.0 g/dL) is associated with improvements in clinically important outcomes.^57^ Recently, the ATTIRE trial was conducted to evaluate whether targeting an increase in the serum albumin level to 3 g/dL or higher with the use of repeated daily infusions of 20% HSA, as compared to standard care, would reduce the incidence of infection, kidney dysfunction, and death among hospitalized patients with decompensated cirrhosis. Throughout the trial, a median of 200 g (interquartile range, 140-280 g) of HSA per patient was administered in the HSA group compared to 20 g (interquartile range, 0-120 g) administered in the standard care group (adjusted mean difference, 143 g; 95% CI, 127-158.2 g). Of the 397 patients in the standard care group, 196 (49.4%) did not receive any HSA. The primary endpoint was a composite of infection from any cause, kidney dysfunction, and death in hospitalized patients between trial day 3 and day 15, the date of discharge (if before day 15), or the date on which the patient was assessed to be medically fit for discharge (if before day 15). In the intention-to-treat analysis, 113 of 380 patients (29.7%) in the HSA group and 120 of 397 patients (30.2%) in the standard care group had a protocol-defined composite primary endpoint event (adjusted OR, 0.98; 95% CI, 0.71-1.33; *P* = 0.87). This small difference between the groups was not significant; the investigators concluded that, in patients hospitalized with decompensated cirrhosis, HSA infusions to increase the albumin level to a target of 3 g/dL or more are not more beneficial than the current standard care in the UK.^58^

From a clinical perspective, correcting albumin based on fluid status is more important than achieving goal albumin levels. HSA use should be guided by functional (volume status and treatment response; Table 2) rather than quantitative endpoints.^57^

### Hypervolemia.

Because it is difficult to identify the optimum HSA dose, the most common risks of HSA administration are pulmonary edema and fluid overload. Pulmonary edema is precipitated by HSA-induced increases in plasma volume, especially when HSA is infused rapidly. The HSA dose and rate of infusion should be adjusted according to the patient’s volume status,^27,40^ which requires evaluation after each HSA dose, including for signs of cardiopulmonary dysfunction and fluid status (eg, blood pressure, pulse, oxygenation, escalating oxygen requirements, respiratory rate, development of peripheral edema, and renal function). Upon the first clinical sign(s) of cardiovascular overload (headache, dyspnea, jugular venous distention, and increased blood pressure), the infusion must be slowed or stopped immediately,^59^ and furosemide can be considered for volume management. Use of HSA must be undertaken with caution in conditions where hypervolemia and its consequences could represent a special risk to the patient, such as in pulmonary hypertension with right heart failure, congestive heart failure, pulmonary edema, and renal and postrenal anuria.^59^ Assessing intravascular volume status by measuring the inferior vena cava diameter and percent collapsibility with inspiration using conventional ultrasound machines or at the bedside using point-of-care ultrasound could be a useful tool in guiding HSA infusion. Pharmacists should work with the bedside provider team to understand the patient’s volume status when evaluating further doses of HSA.

### Sodium content in HSA.

In clinical practice, providers are often unaware of the sodium content in HSA preparations (Table 2), which is included for isotonicity. As a result, hypernatremia occurs in patients administered HSA over several days. The sodium content in albumin is similar per volume to that of normal saline. This high sodium content may contribute to associated pulmonary edema. Pharmacists are essential in educating the multidisciplinary team on this topic.

### Special considerations with HSA and vasoconstrictor coadministration.

In patients with HRS-AKI, the additive effects provided by vasoconstrictors and HSA infusion are thought to improve outcomes when compared to either agent alone,^10,13^ but this may further complicate the adverse event profile. According to AASLD guidance, patients should be closely monitored for the possible development of adverse effects of vasoconstrictors and HSA, including ischemic complications and pulmonary edema.^9^

In the CONFIRM study, which evaluated terlipressin plus HSA compared to placebo, more adverse events, including abdominal pain, nausea, diarrhea, and respiratory failure, occurred with terlipressin than with placebo, and death within 90 days due to respiratory disorders occurred in 11% of patients in the terlipressin group (n = 22) compared to 2% of patients in the placebo group (n = 2).^16^ The adverse respiratory effects observed with terlipressin combined with HSA could be attributed to the increased preload from aggressive hydration with HSA administration. HSA infused concomitantly with terlipressin, which is the current standard of care,^9^ needs to be administered with caution because HSA increases cardiac preload and terlipressin increases afterload, thereby limiting the cardiac reserve of a patient with cirrhosis.^30^ While there is no laboratory value that can determine whether a patient is volume overloaded, using clinical examination, providers should look for signs and symptoms of volume overload in patients (eg, headache, dyspnea, jugular venous distention, increased blood pressure, opacities on chest X-ray, point-of-care ultrasound). In patients with respiratory symptoms attributed to fluid resuscitation, the HSA dose and rate should be adjusted based on the individual patient’s volume status. Cardiac evaluation with echo Doppler should be considered because cardiac dysfunction and pulmonary hypertension are common in advanced cirrhosis.

Norepinephrine plus HSA is the treatment regimen of choice for HRS-AKI when terlipressin is not available. Norepinephrine is often associated with reversible cardiac and digital ischemia.^60^ In addition to the monitoring recommended with vasoconstrictor-plus-HSA coadministration, norepinephrine administration requires intensive hemodynamic monitoring in an intensive care unit setting.^10,61^

Pharmacists can play a key role in adverse event reporting within their institution’s adverse event reporting system. Patient- and drug-specific factors should be captured, such as indication; HSA dose, concentration, frequency, and duration; any concomitant agents (terlipressin or other vasoconstrictors); trend in kidney function (serum creatinine, urinary output, electrolytes); ACLF grade; and any documented measures of volume status listed above. By capturing all of this information and identifying trends, pharmacists can help identify what might have led to the development of the adverse event.

### Long-term HSA administration.

Recently published studies have examined HSA use outside of its current indications, specifically, the efficacy of long-term HSA administration in less acutely ill patients outside hospital settings to modify the natural history of cirrhosis and prevent disease progression.^42^ The ANSWER study is an Italian multicenter, randomized, open-label study examining a population of 431 patients with noncomplicated but persistent ascites despite diuretic administration. The patients were randomized to receive standard medical treatment (SMT) or SMT plus HSA (40 g twice a week for 2 weeks and then 40 g per week) for 18 months. Overall, 18-month survival (the primary endpoint) was significantly higher in the SMT-plus-HSA group than in the SMT group (Kaplan-Meier estimates of 77% vs 66%; *P* = 0.028), indicating that HSA may have the potential to improve survival and, therefore, modify the natural history of decompensated cirrhosis.^62^ On the contrary, the MATCH study randomized 196 patients with cirrhosis and ascites who were awaiting a liver transplant to receive either SMT plus HSA (40 g for 15 days) and midodrine (15-30 mg per day according to pressor response) or SMT plus placebo. The primary endpoint of incidence of any hepatic complication (renal failure, hyponatremia, infections, HE, or gastrointestinal bleeding) was not achieved; there were no significant differences between the groups in the probability of developing complications of cirrhosis during follow-up (*P* = 0.402) or 1-year mortality (*P* = 0.527).^63^ It is postulated that the positive results of the ANSWER study compared to the MATCH study can be attributed to the administration of higher doses of HSA and/or that the patient population was sicker in the MATCH study than in the ANSWER study. As stated by the AASLD, larger, high-quality studies are needed to draw any conclusions on this topic.^9^

## Conclusions

Real-world, multidisciplinary expert perspectives combined with published guidance recommendations presented in this manuscript can help to facilitate HSA use and aid in institutional HSA guideline development for cirrhotic patients with LVP, SBP, and HRS-AKI. Pharmacists can help choose the appropriate concentration of HSA based on indications, which is most often 25% HSA for patients with cirrhosis. They can encourage prompt use in acute situations to help mitigate negative consequences from delays, such as AKI, while also ensuring orders for HSA are reassessed frequently to avoid unnecessary and potentially harmful overadministration. By providing education on the lack of benefit from targeting specific albumin concentrations, pharmacists can help support judicious use of HSA. Lastly, by conducting a thorough literature search for all possible indications where HSA could provide benefit and developing institutional appropriate use criteria, pharmacists can help ensure that consistent recommendations are available for education and to help guide use of HSA. As a key member of the multidisciplinary team, pharmacists can educate peers on these important points, thereby facilitating appropriate HSA use and, ultimately, improving patient care.
